# Activation of the unfolded protein response promotes axonal regeneration after peripheral nerve injury

**DOI:** 10.1038/srep21709

**Published:** 2016-02-24

**Authors:** Maritza Oñate, Alejandra Catenaccio, Gabriela Martínez, Donna Armentano, Geoffrey Parsons, Bredford Kerr, Claudio Hetz, Felipe A. Court

**Affiliations:** 1 Geroscience Center for Brain Health and Metabolism, Santiago, Chile; 2 Millenium Nucleus for Regenerative Biology, Faculty of Biology, Pontificia Universidad Católica de Chile, Santiago, Chile; 3 Biomedical Neuroscience Institute, Faculty of Medicine, University of Chile, Santiago, Chile; 4 Program of Cellular and Molecular Biology, Institute of Biomedical Sciences, Center for Molecular Studies of the Cell, University of Chile, Santiago, Chile; 5 Department of Molecular Biology, Genzyme Corporation, 49 New York Avenue, Framingham, MA 01701, USA; 6 Centro de Estudios Científicos, Valdivia, Chile; 7 Department of Immunology and Infectious diseases, Harvard School of Public Health, Boston MA, USA

## Abstract

Although protein-folding stress at the endoplasmic reticulum (ER) is emerging as a driver of neuronal dysfunction in models of spinal cord injury and neurodegeneration, the contribution of this pathway to peripheral nerve damage remains poorly explored. Here we targeted the unfolded protein response (UPR), an adaptive reaction against ER stress, in mouse models of sciatic nerve injury and found that ablation of the transcription factor XBP1, but not ATF4, significantly delay locomotor recovery. XBP1 deficiency led to decreased macrophage recruitment, a reduction in myelin removal and axonal regeneration. Conversely, overexpression of XBP1s in the nervous system in transgenic mice enhanced locomotor recovery after sciatic nerve crush, associated to an improvement in key pro-regenerative events. To assess the therapeutic potential of UPR manipulation to axonal regeneration, we locally delivered XBP1s or an shRNA targeting this transcription factor to sensory neurons of the dorsal root ganglia using a gene therapy approach and found an enhancement or reduction of axonal regeneration *in vivo*, respectively. Our results demonstrate a functional role of specific components of the ER proteostasis network in the cellular changes associated to regeneration and functional recovery after peripheral nerve injury.

The interruption of an axon is followed by degeneration of the distal segment and a regeneration response in the proximal segment that is fundamental for functional recovery in the peripheral nervous system (PNS). In contrast, damage to axons in the central nervous system (CNS) is followed by poor regeneration and limited functional recovery^1^. Axonal regeneration depends on the intrinsic potential of neurons and the reaction of glial cells which expresses proteins that inhibit or promote axonal regeneration^2^. PNS injury initiates a sequential response known as Wallerian degeneration^3^, characterized by axonal degeneration and dedifferentiation of Schwann cells^4^, which secrete trophic factors and provide the bands of Bungner, cellular alleys that permit axonal growth^5^. In addition, Schwann cells remove axonal and myelin debris and secrete cytokines and chemokines that recruit immune cells to the degenerating nerve to further eliminate cell debris^6^, thus speeding up axonal regeneration^1^. By contrast, axonal damage in the CNS is followed by limited myelin clearance, and reactive astrocytes secrete growth-inhibitory molecules that generate an unfavorable environment for axonal regeneration^7^. Therefore, cellular reactions to stress conditions generated by axonal injury will affect the regenerative capability and therefore functional recovery^2^.

Accumulating evidence indicates that endoplasmic reticulum (ER) proteostasis is altered after injury in both PNS and CNS, generating a protein folding stress reaction in neurons and glial cells^8^. ER stress engages an adaptive reaction known as the unfolded protein response (UPR) that restores cellular proteostasis or trigger apoptosis of damaged cells^9^. The UPR is initiated by activation of specialized sensors including IRE1α, PERK and ATF6. IRE1α catalyzes the unconventional splicing of the mRNA encoding for *Xbp1*, eliminating an intron of 26 nucleotides. This processing event shifts the coding reading frame of the mRNA, leading to the production of an active transcription factor termed XBP1s^9^. XBP1s is essential for the expression of genes involved in protein folding, secretion, phospholipid biosynthesis and ER-associated protein degradation^10^. Activation of PERK inhibits general protein translation through the phosphorylation of eIF2α and allows the selective translation of the transcription factor ATF4, which control the expression of genes involved in redox status, protein metabolism, folding and autophagy^11^. However, under chronic ER stress ATF4 activates pro-apoptotic programs mediated in part by the upregulation of the transcription factor CHOP, the induction of oxidative stress and members of the BCL-2 family^12^. Overall, the UPR integrates information about the nature, intensity and duration of the stress stimulus toward the recovery of proteostasis or the activation of apoptosis.

ER stress is emerging as a relevant condition driving both neurodegeneration and cell survival in the most frequent brain diseases^13^. Mechanical injury to the CNS has also been shown to activate ER stress, having detrimental effects over locomotor recovery^8^. We reported a rapid activation of the UPR after spinal cord injury (SCI) after a few hours that was sustained for several days^14^. Ablation of ATF4 or XBP1 expression reduced locomotor recovery after SCI, whereas administration of an XBP1s-based gene therapy to the injured zone improved oligodendrocyte survival and locomotor recovery^14^. Other studies have also shown a functional effect of the UPR after SCI (see examples in^15^,^16^). Although the upregulation of ER stress markers has been reported in neurons after mechanical damage to the PNS^17^,^18^,^19^,^20^, the functional role of the UPR in axonal regeneration and locomotor recovery after peripheral nerve injury remains to be determined.

Here, we report the activation of an ER stress reaction in the injured nerve, and show that genetic ablation of *Xbp1*, but not *Atf4*, results in a significant delay of locomotor recovery after damage, associated to a decrease in macrophage infiltration, reduced myelin removal and lower density of regenerated axons. Remarkably, overexpression of XBP1s in injured neurons enhanced axonal regeneration *in vivo*. Our results identify selective components of the proteostasis network as possible therapeutic targets to increase locomotor recovery after damage to the nervous system.

## Results

### Peripheral nerve injury triggers a local upregulation of selective UPR responses

**Figure 1:**
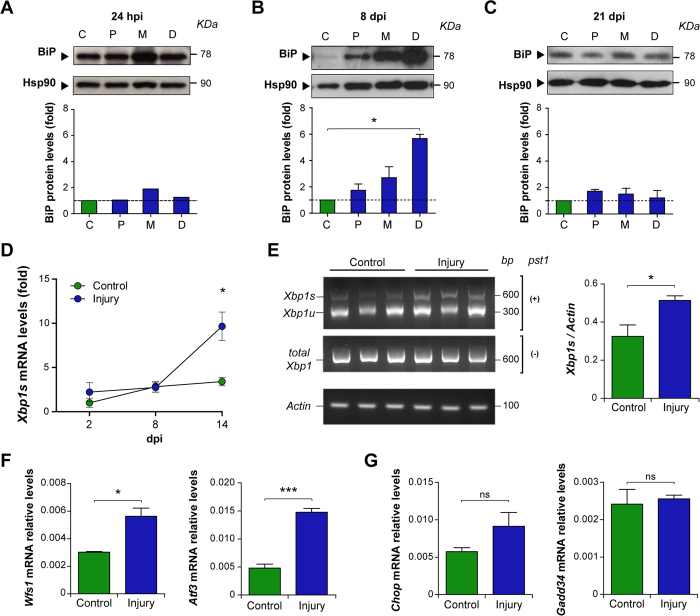
Unfolded protein response is activated after sciatic nerve injury. Wild-type mice were injured and at different days post-injury (dpi) a 5 mm segment of the sciatic nerve was removed in the injured segment (site of injury or middle, M) and proximal (P) and distal (D) for posterior analysis. BiP protein levels were evaluated at **(A)** 24 h post injury (hpi) and at **(B,C)** 8 and 21 days post injury (dpi) in P, M and D segments and compared to contralateral uninjured nerves (label as C). Protein levels were quantified by densitometry and normalized with Hsp90 expression (bottom panel). **(D)** *Xbp1s* mRNA expression was quantified by real-time PCR in D segment at 2, 8 and 14 dpi. **(E)** *Xbp1* spliced (Xbp1s) and unspliced (XBP1u) forms mRNA levels were analyzed in D segment at 14 dpi by RT-PCR followed by PstI digestion. *Actin* levels were used as loading control. **(F)** *Wfs1, Atf3*, **(G)** *Chop and Gadd34*, expression were analyzed from sciatic nerves by real-time PCR in uninjured conditions and at 14 dpi in D segments. Data is expressed as mean ± S.E.M.; *p < 0.05, ***p < 0.001, n.s.: non significant. Statistical differences were analyzed using student’s t-test (n = 3 animals per group).

To characterize the activation of the UPR after nerve crush, we monitored the expression of ER stress-responsive chaperones in sciatic nerve segments containing the crushed area (middle; M), proximal (P) and distal (D) fragments to the injured zone at different times post-injury (see methods). A progressive upregulation of the ER chaperone BiP was detected at early time points after damage using western blot analysis, observing a slight increase at 24 hours post-injury (hpi) in the injured segment compared to the contralateral (C) uninjured nerve (Fig. 1A). At 8 days post-injury (dpi), and compared to uninjured nerves, we detected a peak of BiP expression in the distal segment (Fig. 1B), in which axonal regeneration is taking place^21^. Finally, at 21 dpi, when Wallerian degeneration is complete and remyelination of new fibers is under way, BiP protein levels return to basal values similar to the uninjured condition (Fig. 1C). We also monitored the activation of the ER stress sensor IRE1α, assessed by the quantification of XBP1 mRNA splicing. Kinetic analysis using real time PCR from samples of the distal segment revealed a significant increase in XBP1 mRNA splicing at 14 dpi (Fig. 1D). This result was then corroborated using another XBP1 mRNA assay, using the PstI digestion of a RT-PCR reaction, which resolves both the spliced and unspliced forms (Fig. 1E). In agreement with these results, we observed a significant upregulation of the UPR-target genes *Wfs1*^22^ and *Atf3* at 14 dpi (Fig. 1F). In contrast, analysis of direct markers of the PERK pathway revealed no induction in the mRNA levels of the proapoptotic ATF4 effectors *Chop* and *Gadd34* (Fig. 1G and Supplementary Fig. S1A and B). Similarly, no upregulation of CHOP was observed in sensory and motoneurons using immunohistochemistry (IHC) of dorsal root ganglia (DRG) and the ventral horn of the spinal cord at 8 dpi (Supplementary Fig. S1C).

**Figure 2:**
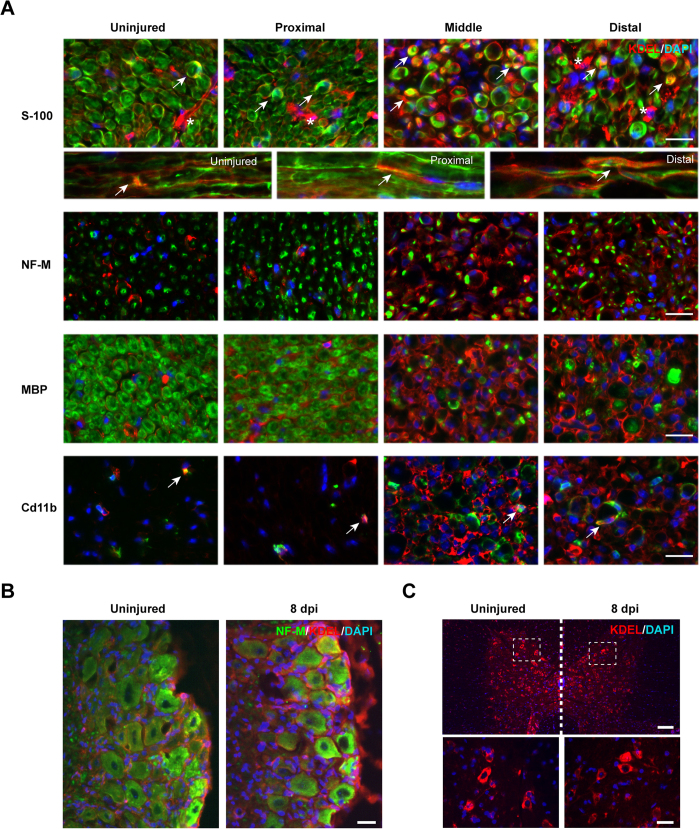
ER stress responses after sciatic nerve crush. Wild-type mice were crush injured in the right sciatic nerve and sham operated in the left sciatic nerve. After 8 days, nerves were removed for histological analysis. **(A)** KDEL staining (red) was performed using indirect immunofluorescence and co-stained with S100 (Schwann cells), NF-M (axons), MBP (myelin), and a Cd11b (macrophages) in green. Cell nuclei were counter stained using DAPI (blue). Co-localization is denoted using white arrows and S100 negative KDEL positive cells with asterisk. Scale bar: 20 μm. **(B)** DRGs and were collected from animals described in A and KDEL staining (red) was performed together with the neuronal marker NF-M (green). Scale bars: 100 μm. **(C)** Thoracic spinal cord from animals described in A was analyzed for KDEL staining (red). Scale bars: 100 μm (low magnification) and 40 μm (high magnification).

To define the cell types engaging an ER stress reaction after sciatic nerve crush, we monitored the expression levels of ER chaperones by immunofluorescence (IF) using an anti-KDEL antibody that mostly recognizes BiP and Grp94. A marked increase in KDEL staining was observed in crushed nerves, and more markedly in middle and distal segments compared with control tissue (Fig. 2A). Interestingly, the induction of ER stress in peripheral nerves was mostly observed in Schwann cells and not in axons as revealed after co-staining of KDEL with anti-S100, MBP or neurofilament medium chain (NF-M) antibodies, respectively (Fig. 2A). Infiltrating macrophages (CD11b positive) showed only a slight increase in KDEL staining after nerve crush (Fig. 2A). In addition, the expression of ER stress markers was confirmed in neuronal cell bodies in DRGs (Fig. 2B), as previously reported^19^, whereas spinal cord motoneurons did not show signs of increased KDEL staining (Fig. 2C), Taken together, our results support the occurrence of an ER stress reaction at the neuronal cell bodies and in Schwann cells at the distal segment of the peripheral nerve undergoing degeneration and regeneration, and involving the specific activation of the IRE1α/XBP1 axis.

### XBP1 deficiency in the nervous system decreases locomotor recovery and nerve changes associated to Wallerian degeneration

**Figure 3:**
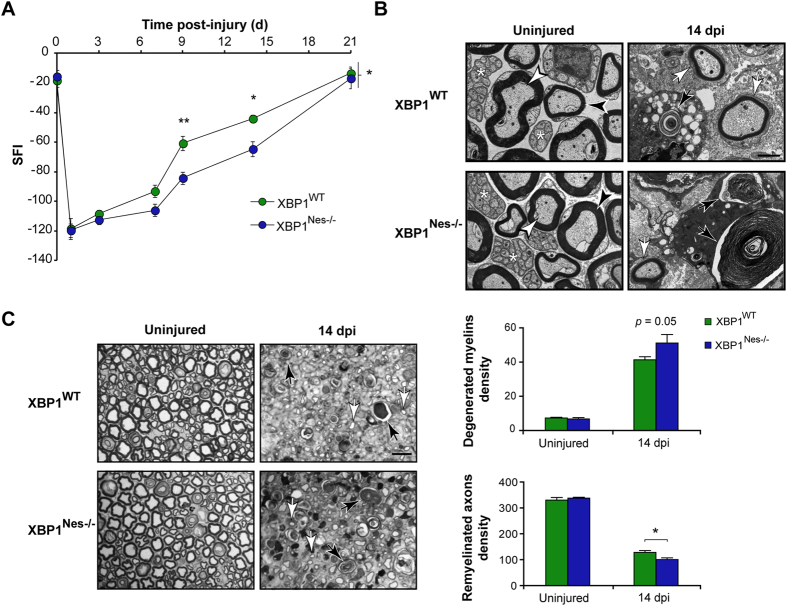
XBP1 deficiency decreases myelin removal, axonal regeneration and locomotor recovery after sciatic nerve injury. **(A)** XBP1^WT^ and XBP1^Nes−/−^ mice were crush injured in the right sciatic nerve and sham operated in the left sciatic nerve. Locomotor recovery was evaluated using the SFI analysis before (0 day) and at indicated time points. **(B)** Electron microscopy of uninjured and distal segments of 14 dpi from XBP1^WT^ and XBP1^Nes−/−^ mice. White arrowheads indicate intact myelinated axons, black arrowheads, intact myelins, and asterisks unmyelinated axons. Black arrows point to degenerated myelins and white arrows, to remyelinated axons. Scale bar: 2 μm. **(C)** Transversal semi-thin sections of sciatic nerves stained with toluidine blue from uninjured and at 14 dpi XBP1^WT^ and XBP1^Nes−/−^ mice. Sections were obtained 3 mm distal to crush segment. Black and white arrows indicate demyelinated and regenerated fibers, respectively (left panel). Scale bar: 10 μm. Quantification of degenerated myelins and remyelinated axons density is presented (right panel). Data are shown as mean ± S.E.M.; *p < 0.05; **p < 0.01. SFI data were analyzed by repeated measures ANOVA followed by Bonferroni’s post hoc test (n = 7 animals per group). Morphological data was analyzed at each time point by student’s t-test (n = 3 animals per group).

To define the functional relevance of the UPR to nerve degeneration and regeneration we performed genetic manipulation of two key UPR transcription factors in mice. First, Nestin-Cre LoxP system was used to specifically ablate XBP1 expression in the nervous system (XBP1^Nes−/−^), a mouse model we have previously characterized^23^. The expression of Cre and the deletion of exon II of the *Xbp1* gene were confirmed in DRGs and the sciatic nerve using real time PCR (Supplementary Fig. S2A and S2B). After sciatic nerve crush, locomotor performance was estimated using the sciatic nerve functional index (SFI, see Methods). XBP1^Nes−/−^ mice presented a delay in locomotor recovery following nerve crush compared with XBP1^WT^ littermates (Fig. 3A). The progression in locomotor performance was significantly different between genotypes at intermediate stages of recovery, but in both strains a complete recovery was reached at 21 dpi.

The morphological changes associated with nerve repair were studied under the electron microscope (EM) in the distal segment. Since a significant difference in locomotor recovery was found at 14 dpi, we used this time point for the histological analysis. XBP1^Nes−/−^ nerves presented more myelin debris (Fig. 3B, black arrows) and fewer remyelinated fibers density compared with XBP1^WT^ mice (Fig. 3B, white arrows). Quantification of these parameters in semi-thin sections at the light microscope, revealed a barely greater density of degenerating myelins (51.2 ± 4.9 vs. 41.3 ± 1.8) and a significant reduction in remyelinated axons in XBP1^Nes−/−^ mice compared with XBP1^WT^ nerves (100.7 ± 6.5 vs. 128.3 ± 6.7, Fig. 3C). Together these results suggest that XBP1 expression modulates nerve repair after crush.

### ATF4 does not contribute to locomotor recovery and axonal regeneration in peripheral nerves

**Figure 4:**
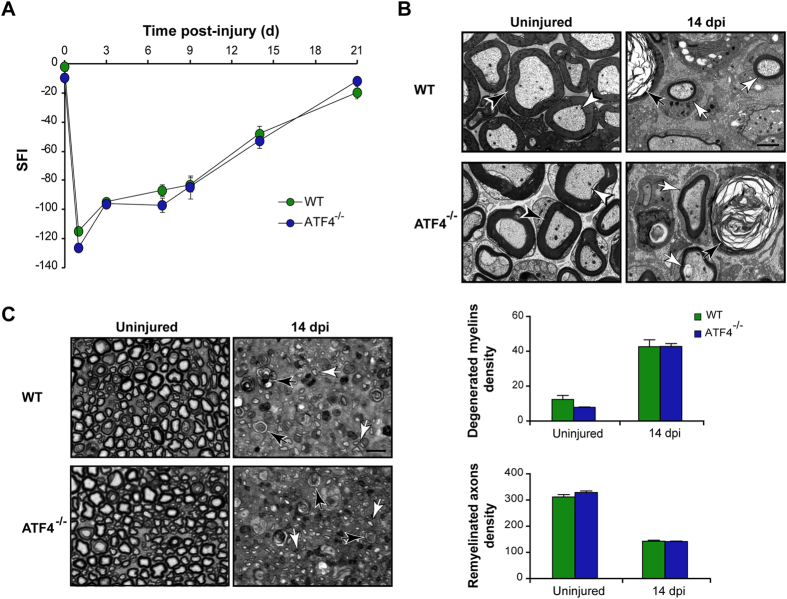
ATF4 deficiency does not affect axonal regeneration or locomotor recovery after sciatic nerve injury. **(A)** Wild-type (WT) and ATF4^−/−^ mice were crush injured in the right sciatic nerve and sham operated in the left sciatic nerve. Locomotor recovery was evaluated using the SFI analysis before (0 day) and at indicated time points. **(B)** Electron microscopy of uninjured and distal segments of 14 dpi from WT and ATF4^−/−^ mice. White arrowheads indicate intact myelinated axons and black arrowheads, intact myelins. Black arrows point to degenerated myelins and white arrows, to remyelinated axons. Scale bar: 2 μm. **(C)** Transversal semi-thin sections of sciatic nerves stained with toluidine blue from uninjured and at 14 dpi WT and ATF4^−/−^ mice. Sections were obtained 3 mm distal to crush segment. Black and white arrows indicate demyelinated and regenerated fibers, respectively (left panel). Scale bar: 10 μm. Quantification of degenerated myelins and remyelinated axons density is presented (right panel). Data are shown as mean ± S.E.M.; SFI data were analyzed by repeated measures ANOVA followed by Bonferroni’s post hoc test (n = 7 animals per group). Morphological data was analyzed at each time point by student’s t-test (n = 3 animals per group).

To define if the effects of XBP1 were specific to this UPR branch, we next assessed the contribution of ATF4 to locomotor recovery after nerve damage. To this end, ATF4 full knockout (ATF4^−/−^) mice^24^ were subjected to a sciatic nerve crush and motor performance was monitored. Surprisingly, the locomotor recovery of ATF4^−/−^ and control wild-type animals was virtually identical at all time points analyzed, reaching full recovery at 21 dpi (Fig. 4A). The EM study of nerves in the distal segment at 14 dpi revealed that the clearance of myelin debris (42.7 ± 3.9 vs. 42.9 ± 1.6) and remyelination of regenerating axons (142.4 ± 4.0 vs. 141.6 ± 1.7) was similar in ATF4^−/−^ and control nerves (Fig. 4B,C). These results are consistent with the poor induction of ATF4 target genes (Fig. 1G and Supplementary Fig. S1). Taken together with our previous results, these observations suggest that the XBP1-UPR branch is specifically activated after nerve injury, impacting axonal regeneration and locomotor recovery.

### XBP1s overexpression accelerates axonal regeneration and locomotor recovery after peripheral nerve injury

**Figure 5:**
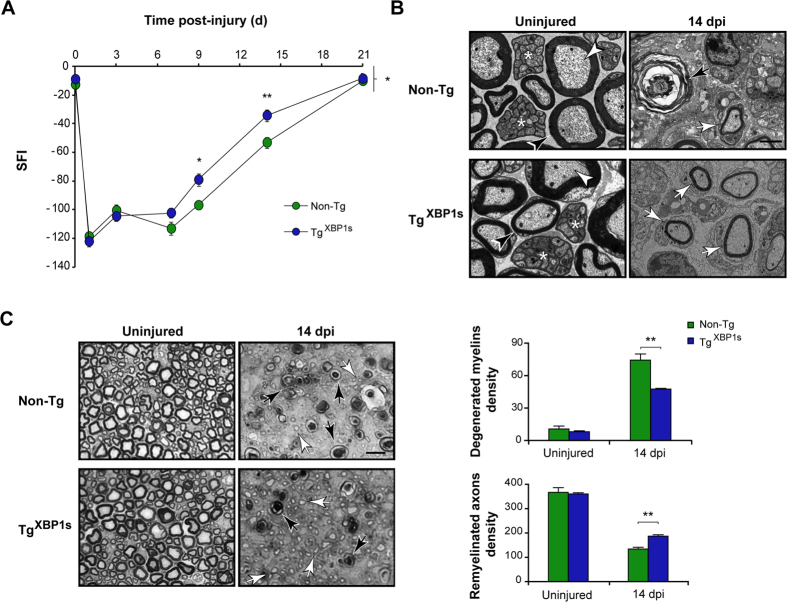
Overexpression of XBP1s increases myelin removal, axonal regeneration and locomotor recovery after peripheral nerve injury. (**A**) Tg^XBP1s^ and Non-Tg mice were damaged in the right sciatic nerve and sham operated in the left side. Locomotor performance was evaluated using the SFI before at the indicated time points. (**B**) Uninjured and distal segments of 14 dpi from Tg^XBP1s^ and Non-Tg mice were analyzed by EM. In control conditions black arrowheads indicate compact myelin, white arrowheads, myelinated axons and asterisk, unmyelinated axons. At 14 dpi, white and black arrows point remyelinated fibers and degenerated myelins, respectively. Scale bar: 2 μm. (**C**). Tg^XBP1s^ and Non-Tg sciatic nerves from uninjured and at 14 dpi transverse semi-thin sections were obtained 3 mm distal to the crush segment and stained with toluidine blue. Black and white arrows indicate demyelinated and regenerated fibers, respectively (left panel). Scale bar: 10 μm. Quantification of the density of degenerated myelins and remyelinated axons was performed from transverse semi-thin sections of each mouse strain (right panel). Data are shown as mean ± S.E.M.; *p < 0.05; **p < 0.01. SFI data were analyzed by repeated measures ANOVA followed by Bonferroni’s post hoc test (n = 7 animals per group). Morphological data was analyzed at each time point by student’s t-test (n = 3 animals per group).

We raised the working hypothesis that upregulation of UPR-transcriptional responses in neurons and Schwann cells reduce ER stress thus accelerating axonal regeneration and functional recovery. To this end, we used a recently generated mouse model that overexpresses spliced XBP1 under the control of the prion promoter (Tg^XBP1s^)^25^. These animals are viable and born at a Mendelian rate, and did not show any motor phenotype as monitored with the rotarod assay (data not shown). We confirmed the overexpression of XBP1s in DRGs, cerebellum and sciatic nerve using real time PCR to measure *Xbp1s* expression levels in central and peripheral neurons and Schwann cells (Supplementary Fig. S2C). First, we evaluated locomotor recovery after nerve crush in Tg^XBP1s^ and non-transgenic littermates (Non-Tg). Remarkably, SFI analysis revealed that Tg^XBP1s^ mice have a significant increase in locomotor recovery compared to control mice (Fig. 5A). Consistent with these results, Tg^XBP1s^ nerves exhibited a reduction in the density of degenerated myelins (74.2 ± 5.7 vs. 47.5 ± 0.6) and augmented density of remyelinated axons (134.1 ± 7.3 vs. 187.3 ± 5.1) compared to control sciatic nerves at 14 dpi (Fig. 5B,C). Similar results were obtained when Tg^XBP1s^ animals were analyzed at 11 dpi (Supplementary Fig. S3A). Importantly, analysis of the g-ratio in both XBP1^Nes−/−^ and Tg^XBP1s^ nerves indicated that remyelination efficiency was similar when compared with control wild-type littermates (Supplementary Fig. S4), indicating that XBP1 expression modulates the velocity of axonal regeneration rather than the myelination process. These results suggest that artificial enforcement of XBP1s in the nervous system increases the regenerative capacity of injured peripheral nerves.

### XBP1 expression enhances macrophage infiltration and MCP-1 expression

**Figure 6:**
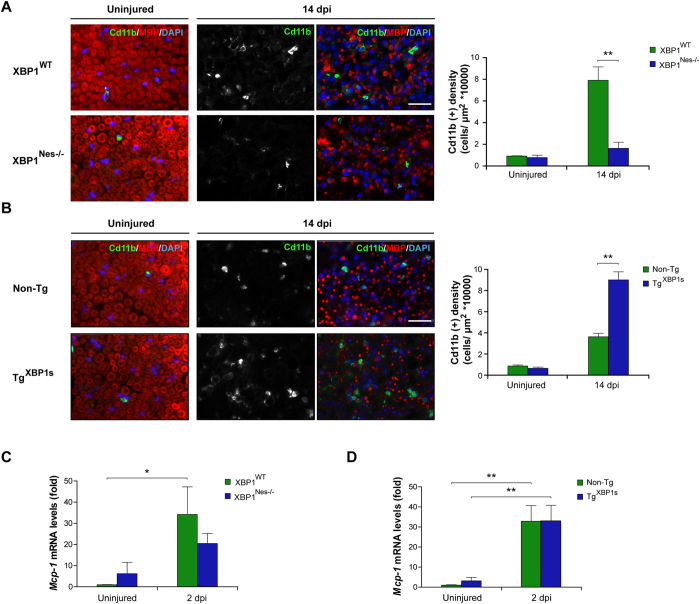
XBP1 expression in the nervous system enhances macrophage infiltration in injured sciatic nerves. **(A)** Sciatic nerves from XBP1^Nes−/−^ and XBP1^WT^ littermates were processed for immunofluorescence from uninjured conditions and at 14 dpi distal sciatic nerves were analyzed for Cd11b (green) to evaluate macrophages and MBP (red) to stain myelin sheaths. Nuclei were counterstained using DAPI (blue, left panel). The staining density for Cd11b was quantified at 14 dpi in XBP1^Nes−/−^ and XBP1^WT^ mice (right panel). **(B)** Tg^XBP1s^ and non-Tg sciatic nerves were analyzed as described in A. *Mcp-1* expression was analyzed in sciatic nerves of XBP1^Nes−/−^ and XBP1^WT^ mice **(C)** or in Tg^XBP1s^ and non-Tg sciatic nerves **(D)** by real-time PCR at 2 dpi. Data are shown as mean ± S.E.M.; *p < 0.05; **p < 0.01. Data were analyzed by student’s t-test at each time point (n = 3 animals per group). Scale bar: 20 μm.

Macrophages participate in the removal of myelin debris, thus contributing to nerve repair. The effect of XBP1 upon the recruitment of Cd11b-positive (Cd11b^+^) macrophages was assessed in crushed nerves with altered XBP1 expression. A significant increase in the density of Cd11b^+^ macrophages was observed in XBP1^WT^ but not in XBP1^Nes−/−^ mice after 14 dpi (7.9 ± 1.2 vs. 1.6 ± 0.6) (Fig. 6A). Importantly, in XBP1^Nes−/−^ mice macrophage density was comparable to the density measured in wild-type animals at basal levels (uninjured condition). We then analyzed the extent of macrophage infiltration by measuring Cd11b^+^ density in Tg^XBP1s^ mice. Consistent with our previous results, analysis of Tg^XBP1s^ mice revealed a significant enhancement in macrophage recruitment compared to Non-Tg littermates at 14 dpi (9.0 ± 0.8 vs. 3.6 ± 0.3) (Fig. 6B). Similar results were observed when an earlier time point was analyzed at 5 dpi (12.5 ± 0.5 vs. 9.4 ± 0.5) (Supplementary Fig. S3B). As these quantifications were expressed as macrophage density, we monitored the area of the nerve to control for possible alterations in its volume due to edema, observing no effects upon manipulation of XBP1 levels (Supplementary Fig. S5).

One of the major signals involved in the infiltration of macrophages to the injured area is the production of the chemokine MCP-1^26^. We monitored the mRNA levels of *Mcp-1* in crushed nerves using real time PCR, and observed a clear upregulation in damaged animals at 2 dpi (Fig. 6C,D). Interestingly, analysis of XBP1^Nes−/−^ animals indicated a reduction in the expression of *Mcp-1* in crushed nerved when compared to littermate controls (Fig. 6C). In contrast, at this time point, no effects were observed in Tg^XBP1s^ mice possibly because *Mcp-1* expression was at its maximun value already saturated (Fig. 6D). Taken together, these results suggest that the effects of XBP1 expression on myelin removal and axonal regeneration correlate with the enhancement of *Mcp-1* expression and macrophage infiltration to the injured nerves.

### Local XBP1s gene transfer to DRGs enhances axonal regeneration after peripheral nerve damage

**Figure 7:**
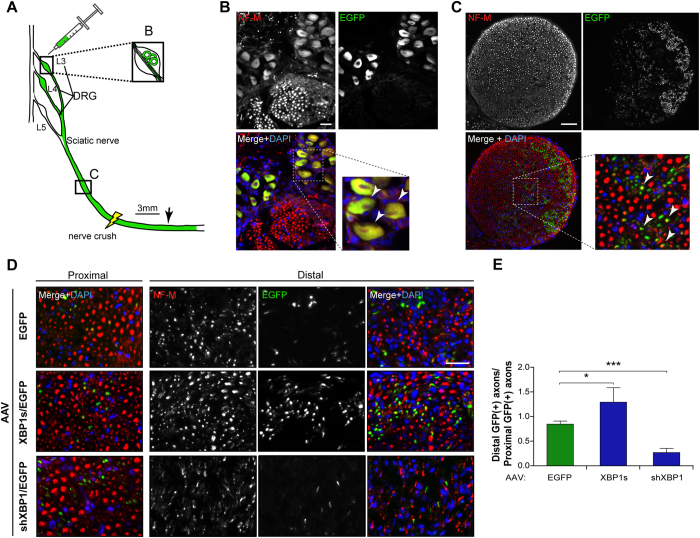
XBP1s overexpression in neurons enhances axonal regeneration *in vivo*. **(A)** Schematic representation of the experimental design. Wild-type mice were injected with 1μl of AAV EGFP, AAV XBP1s/EGFP or AAV shXBP1/EGFP in L3 and L4 DRGs. EGFP expression was used to identify transduced neurons. At 7 days post-injection the sciatic nerve was crushed at the sciatic notch level (yellow light bolt) and at 14 dpi the nerves were dissected and analyzed in transverse sections 3 mm distal to the injury (black arrow) and normalized to EGFP-positive axons 6 mm proximal to the crush (C-labeled box). **(B)** EGFP expression (green) of infected neurons from DRGs, 7 days after injection in uninjured nerves. NF-M immunostaining (red) was used to identify neuronal somas and axons. Scale bar: 100 μm. **(C)** Cross section of an uninjured nerve 7 days after AAV injection. EGFP fluorescence in green, immunostained for NF-M (red) and counterstained using DAPI (blue). Transduced somas and axons are indicated with white arrowheads in the insets of B and C. Scale bar: 100 μm. **(D)** Axonal regeneration in the distal sciatic nerve was evaluated in AAV-EGFP (upper panel), AAV-XBP1/EGFP (middle panel) and AAV-shXBP1/EGFP (lower panel) injected DRGs at 14 dpi. Scale bar: 20 μm. **(E)** Quantification of mean EGFP^+^/NF-M^+^ axons in distal segment normalized to proximal EGFP^+^/NF-M^+^ axons of the same sciatic nerve from AAV-EGFP, AAV-XBP1s/EGFP and AAV-shXBP1/EGFP injected mice. Data are expressed as mean ± S.E.M. *p < 0.05. Student’s t-test was performed for statistical analysis against control EGFP condition (n = 3 per condition).

Our previous results are indicative of a functional role of XBP1 in the regenerative response observed in the PNS after nerve injury. Both mouse models used to perform XBP1 gain- and loss-of-function target neurons and possibly other cell types, including glial cells, and the genetic manipulation is active since embryonic development. To assess a possible cell-autonomous contribution of XBP1 to axonal regeneration, we evaluated the consequences of delivering an active form of XBP1 selectively into neurons of adult animals. We developed serotype 2 Adeno-Associated Viruses (AAV) to overexpress XBP1s in sensory neurons in addition to green fluorescent protein (EGFP) to identify infected cell bodies and axons as we reported^14^,^27^. Neurons of L3 and L4 DRGs are the principal contributors which project their axons into the sciatic nerve^28^ (Fig. 7A). In 2 month-old mice, these ganglia were injected with AAVs to deliver XBP1s (AAV XBP1s/EGFP) or EGFP alone (AAV EGFP). The efficiency of viral transduction was confirmed by inspection of EGFP signal 7 days after AAV injection, observing that both neuronal somata at DRGs and sciatic nerve axons were positive for EGFP (Fig. 7B,C).

Then, the sciatic nerve was crushed and regenerated EGFP-positive axons were quantified in the distal nerve stump at 14 dpi. Axonal regeneration was evaluated by quantifying the number of EGFP-positive axons co-labeled with NF-M in cross sections at 3 mm distal to the crush segment (Fig. 7D). Regenerated axons were normalized to the number of EGFP- and NF-M-double positive axons at 6 mm proximal to the crush segment of the same nerve, to control for possible differences in AAV-transduction efficiency between animals. Remarkably, we observed a significant 1.5 fold increase in the number of double-positive regenerated axons when XBP1s was expressed in comparison to control axons expressing EGFP alone (Fig. 7E). To target the expression of endogenous XBP1 locally in sensory neurons, we then performed a similar experiment by delivering an shRNA against XBP1 using an AAV construct we recently characterized *in vivo*^27^. In agreement with our previous experiments, knocking down Xbp1 significantly reduced the number of regenerated axons compared with control AAV-GFP vector (Fig. 7D,E). These results further support the concept that XBP1 expression in neurons enhances the intrinsic regenerative capabilities after peripheral nerve damage.

## Discussion

Alteration to the proteostasis network is emerging as a relevant player in the most common neurodegenerative diseases that involve protein misfolding and aggregation^13^. Evidence of failure in organelle function in neurons, in particular the ER and mitochondria, has been extensively described after axotomy both in the neuronal cell body and its axon. We have recently reported that local release of calcium from the ER^29^ and the activation of the mitochondrial permeability transition pore are key events in the axonal degeneration program^30^,^31^.

Although extensive reports are available describing the impact of the UPR to neuronal degeneration of the soma, no studies have determined the functional impact of the UPR to axonal degeneration and regeneration. Only a few correlative reports have associated mechanical injury in the PNS with the occurrence of ER stress in neurons projecting to the damaged nerve as well as in Schwann cells^18^,^19^,^31^. Interestingly, a recent report suggested that the global deregulation of miRNAs in damaged sciatic nerve axons may influence the global ER stress response^32^. Furthermore, in models of Charcot-Marie-Tooth-1B disease, a pathology involving the misfolding and ER-retention of a mutated form of the P0 myelin glycoprotein, genetic targeting of *Chop* fully rescues motor deficits, reducing demyelination^33^,^34^. Moreover, a recent study demonstrated that UPR target genes are upregulated in soma and axons after nerve injury and that BiP and CHOP are retrogradelly transported from injured axons toward neuronal cell bodies^20^. Depending on the duration and intensity of the stress stimuli the UPR has dual roles in both cell survival (adaptive phase) and cell death (terminal UPR)^9^. Thus, functional studies are needed to define the contribution of this pathway to peripheral nerve injury. Here, we confirmed a progressive activation of the UPR after sciatic nerve crush, correlating with the kinetics of Wallerian degeneration. Importantly, we showed for the first time a functional and selective role of the IRE1α/XBP1 pathway in locomotor recovery and axonal regeneration after sciatic nerve injury using genetic approaches. In agreement with these findings, we recently reported that overexpression of the ER foldase ERp57/Grp58 in neurons using transgenic mice also enhances axonal regeneration and locomotor recovery after peripheral nerve crush^17^, indicating that the ER proteostasis network is a relevant contributor to the axonal repair program.

The pro-regenerative activity of XBP1 under conditions of nerve damage was associated to remodeling events in the nerve during Wallerian degeneration, including improved myelin removal and macrophage infiltration. Unexpectedly, genetic ablation of ATF4 had no effects in myelin removal, axonal regeneration and locomotor recovery, suggesting a specific impact of XBP1 in axonal regeneration. Consistent with these findings, previous evidence revealed that eIF2α phosphorylation and CHOP expression are repressed in dedifferentiating Schwann cells following nerve injury^31^. We also observed that although XBP1 mRNA splicing was significantly induced after nerve crush, *Chop* and *Gadd34* remained unchanged. In contrast, *in vitro* studies using *Chop* deficient Schwann cells revealed an important proapoptotic activity of this factor upon proinflammatory challenges^31^ and in Charcot-Marie-Tooth-1B disease^33^,^34^. Finally, another study indicated that sciatic nerve injury does not activate the PERK signaling branch in motoneurons with injured axons^19^. Therefore, based on these reports and the systematic comparison presented here using XBP1 and ATF4 deficient animals, we speculate that selective activation of the IRE1α pathway results in successful regeneration and locomotor recovery after neuronal damage. Since retrograde injury signals contribute to the transition of the neuron into a regenerative state^35^, local activation in the axonal compartment of IRE1α and XBP1 mRNA splicing may provide an axonal-ER signal proximal to the site of nerve injury to engage the neuron into a regenerative program. A similar concept was recently reported for the ATF6 family member LUMAN/CREB3^20^,^36^. Local cleavage of LUMAN after axonal injury leads to the retrograde transport of the basic leucine zipper transcription factor domain to the nucleus, contributing to axonal regeneration^36^. Importantly, LUMAN also controls ER stress genes. Moreover, a recent report indicated that knocking down LUMAN delays axonal regeneration of sensory neurons^20^. Since XBP1s and ATF6 heterodimerizes to control a subset of UPR target genes^37^, and ATF6 can upregulate XBP1 mRNA^38^, it will be interesting to study if both LUMAN and XBP1s cooperatively modulate gene expression programs associated to axonal regeneration.

Accumulating evidence in models of neurodegeneration support the idea that the contribution of each UPR signaling branch to the disease process is complex and highly dependent on the disease-triggering mechanisms and the cell type affected^13^. For example, we have shown that targeting either XBP1 or ATF4 in SCI models actually diminishes locomotor recovery after mechanical damage to the spine, possibly associated with reduced oligodendrocyte survival^14^. Besides, other reports have shown that expression of CHOP increases tissue damage after SCI^15^. In contrast, XBP1 deficiency does not have an effect after optic nerve injury upon retinal neurons whereas CHOP expression operates as a relevant proapoptotic factor in retinal neurons^39^. In ALS, XBP1 or ATF4 deficiency are protective through the control of autophagy and apoptosis, respectively^40^,^41^, whereas in Huntington’s disease only XBP1 deficiency has beneficial effects^42^. In contrast, in prion related disorders XBP1 is irrelevant^23^, whereas PERK has a pro-degenerative activity^43^. Based on these results, we speculate that the distinctive UPR responses triggered in the PNS versus the CNS might help explaining the differential regenerative capabilities of each nervous system compartments.

The current study suggests that XBP1 expression may have two functional consequences during axonal degeneration and regeneration processes: a cell-autonomous effect in the neuron enhancing axonal regeneration, and a remodeling activity of the nerve microenvironment possibly associated with Schwann cell dedifferentiation and myelin removal. The pro-regenerative mechanisms associated to IRE1α/XBP1 signaling after nerve injury remains to be determined. Importantly, we recently uncovered a novel function of XBP1 in the physiology of the nervous system, controlling synaptic plasticity and learning and memory^25^. Unexpectedly, this function of XBP1 was ER stress-independent and mediated by the direct control in the expression of brain-derived neurotrophic factor (BDNF)^25^. Since BDNF has been shown to control neuronal survival and regeneration^44^, its contribution to the effects of XBP1 to axonal regeneration remains to be determined.

Our results cannot completely discard an additional effect over axonal sprouting, nevertheless the increase or decrease in remyelinated fibers after XBP1s overexpression or in *Xbp1* ablated mice, respectively, suggest an increase in the efficiency of regeneration, by either modifying initiation of the process or by changing the regenerative capacity of the neuron. Thus, based on the complementary evidence provided in this study, we speculate that the effects of XBP1 expression on the locomotor recovery after peripheral nerve damage are due to direct modulation of axonal regeneration programs and not associated to the survival of sensory or motor neurons, or axonal sprouting. Although the gene therapy approach to deliver active XBP1s into DRG neurons demonstrate an effect over axonal regeneration, no behavioral tests were performed as only a small fraction of all sensory neurons were transduced, which is not expected to generate a change in the locomotor parameters studied.

Interestingly, the importance of the UPR in other tissues also involves activities beyond the control of protein folding, quality control and phospholipid synthesis, where this pathway modulates cell differentiation and dedifferentiation programs^45^, a phenomena we suggest will be relevant also in the context of Schwann cell biology. XBP1 expression is fundamental for the differentiation of many distinct cell types, including B lymphocytes^46^,^47^, zymogenic cells in the gastric epithelium^48^, exocrine pancreas and salivary glands^49^, among other cell types^50^. In chondrocytes, ER stress reprogram the cell towards a dedifferentiation process into non-secreting cells, probably operating as a protective response^51^; and a similar concept was recently demonstrated in cancer models^52^,^53^. Differentiated myelin-forming Schwann cells produce massive amounts of lipids and proteins, with transmembrane proteins trafficking through the ER accounting for near 20–50% of total proteins^54^. Based on this fundamental aspect of Schwann cell physiology, it is feasible to propose that this specific cell type may be more prompt to undergo ER stress after sciatic nerve damage. In agreement with this concept, ER stress has been extensively reported to drive oligodendrocyte death in models of multiple sclerosis^55^. The UPR, and more specifically XBP1, has also a relevant function in the innate immune system, controlling cytokine production in macrophages^56^. Both IL-6 and MCP-1 are expressed by dedifferentiated Schwann cells and are involved in macrophage recruitment^57^. Our results after XBP1 ablation, demonstrate a correlation between reduction in *Mcp-1* expression, decreased macrophage recruitment and locomotor recovery after nerve injury, which suggest a contribution of the UPR to the inflammatory microenvironment in the damaged PNS. Overall, our results demonstrate for the first time a functional impact of the UPR in Wallerian degeneration, modulating axonal regeneration and locomotor recovery. Since new small molecules and gene therapy strategies are available to target the UPR^58^, manipulation of the ER proteostasis network might emerge as a new avenue for future therapeutic intervention to improve axonal regeneration.

## Materials and Methods

### Experimental animals

C57BL6 mice weighting between 20 and 25 g were kept under standard conditions of light and temperature, with food and water *ad libitum*. XBP1 floxed mice and ATF4 knockout mice has been previously described^23^,^24^. Briefly, for the generation of XBP1^Nes−/−^ mice, XBP1^flox/flox^ mice were crossed with animals expressing the Cre recombinase under the control of the Nestin promoter to specifically delete *Xbp1* in the nervous system. ATF4^−/−^ mice were generated by crossing heterozygous animals.

### Generation of XBP1s transgenic mice

The mouse XBP1s cDNA was subcloned into the MoPrP.Xho vector^59^ to drive XBP1s expression by the PrP promoter. The presence of the transgene was confirmed by PCR of genomic DNA from the tail (forward primer: 5′-ACACGCTTGGGAATGGACAC-3′; reverse: 5′-CCATGGGAAGATGTTCTGGG-3′). Line 3 was used based on the intensity and tissue-specificity of the expression of the transgene. Transgenic mice were generated at the mouse facility of the Centro de Estudios Científicos, Valdivia, Chile^25^.

### Surgical procedures

Surgical procedures were carried out under 330 mg/Kg of 2-2-2 tribromoethanol (Sigma,) anesthesia and with 30 mg/Kg of Tramadol as analgesic. For sciatic nerve injury, the right nerve was exposed at the level of the sciatic notch and then it was crushed three times for 5 seconds with Dumont #5 forceps (Fine Science Tools INC.). Graphite powder applied to the tip of the forceps before nerve crush was used to identify the crush site when tissue was dissected. The left sciatic nerve was used as a sham operated control group. For induction of ER stress in the substantia nigra, mice received a single intracerebral injection of 2 μL of tunicamycin (5 μg/μL) at the following coordinates: AP: −0.29 cm ML: −0.13 cm and DV: −0.42 cm for 24 hours. Experiments with animals followed protocols approved by the Institutional Animal Care and Use Committees and complied with National Institutes of Health guidelines.

### RNA extraction and RT-PCR

A 5 mm segment of the sciatic nerve obtained 3 mm distal to injury (identified by the graphite stained tissue), DRGs from L3 and L4 spinal nerves and cerebellum were collected and homogenized in Trizol (Invitrogene) for total RNA extraction using standard protocols. cDNA was synthesized with a High Capacity cDNA Reverse Transcription kit (Applied Biosystems). Quantitative real time PCR was performed using SYBR Green fluorescent reagent and an Mx3005P QPCR System (Strategene) and the following primers: *Xbp1s* (forward: 5′-TGCTGAGTCCGCAGCAGGTG-3′, reverse: 5′-GACTAGCAGACTCTGGGGAAG-3′), *Wfs1* (forward: 5′-CCATCAACATGCTCCCGTTC-3′, reverse: 5′- GGGTAGGCCTCGCCAT-3′), *Atf3* (forward: 5′-TTGACGGTAACTGACTCCAGC-3′, reverse: 5′-GAGGATTTTGCTAACCTGACACC-3′), *Chop* (forward: 5′-TGGAGAGCGAGGGCTTTG-3′, reverse: 5′-GTCCCTAGCTTGGCTGACAGA-3′), *Gadd34* (forward: 5′-TTACCAGAGACAGGGGTAGGT-3′, reverse: 5′-GAGGGACGCCCACAACTTC-3´), *Cre* (forward: 5′-ATCGCTCGACCAGTTTAGTT-3′, reverse: 5′-CTGACGGTGGGAGAATGTTA-3′), *Xbp1*Δ (forward: 5′-CCTGAGCCCGGAGGAGAA-3′, reverse: 5′-CTCGAGCAGTCTGCGCTG-3′), *Mcp-1* (forward: 5′-GTCCCTGTCATGCTTCTGG-3′, reverse: 5′-GCGTTAACTGCATCTGGCT-3′) and *β-Actin* (forward: 5′-CTCAGGAGGAGCAATGATCTTGAT-3′, reverse: 5′-TACCACCATGTACCCAGGCA-3′). The relative amount of mRNA was calculated by the comparative threshold cycle method with *β-Actin* as control. Primer sequences were obtained from PrimerBank. *Xbp1* mRNA splicing assay was performed using PstI digestion of PCR products as previously described^60^ using the following primers: (forward 5′-AAACAGAGTAGCAGCGCAGACTGC-3′, reverse 5′-GGATCTCTAAAACTAGAGGCTTGGTG-3′).

### Western blot analysis

A 5 mm sciatic nerve segment containing the injury site (middle), and proximal and distal nerve segments of the same size were collected and homogenized using a plastic Dounce homogenizer in extraction buffer (95 mM NaCl, 25 mM Tris-HCl pH 7.4, 10 mM EDTA pH 8.0, 1% SDS, 1 mM NaF, 1 mM Na_3_VO_4_ and 1% Protease Inhibitor Cocktail (PIC, Sigma-Aldrich, #P8340). Lysates were sonicated, centrifuged and the supernatant was used for protein analysis. Western blot was performed using SDS-PAGE and polyvinylidene difluoride (PVDF) membranes as previously described^30^. The following antibodies were used: anti-HSP90, 1:5000 (Sc-7947, H114, Santa Cruz) and anti-BIP, 1:1000 (SPA-826, Stressgen). Band intensities were normalized to Hsp90 used as a loading control for the nerve lysates. Densitometry analysis was performed using ImageJ software (NIH).

### Locomotor function analysis

Locomotor recovery was evaluated using the sciatic functional index (SFI) as previously described^61^. Paw prints were obtained by moistening the hindlimbs of each animal with black ink and having them walk unassisted along an 11 × 56 cm white paper corridor. Tracks were obtained before surgery (day 0), and 1, 3, 7, 9, 14 and 21 days after nerve injury. The tracks were evaluated for two different parameters: toe spread (TS), the distance between the first and fifth toes, and print length (PL), the distance between the third toe and the hindpaw. Measurements of all parameters were made for the right injured paw (experimental; E) and the left control paw (normal; N) and the SFI was calculated according to the following formula: SFI = 11.89 ((ETS –NTS) / NTS) – 51.2 ((EPL – NPL) / NPL) −7.5.

### Histological analysis

Sciatic nerves were extracted and 3 mm segments located in the injury region (middle), proximal and distal were removed for IF and IHC analysis; a contiguous 3 mm segment located 6 mm distal to the crush site was removed for EM. For IF and IHC analysis, animals were anesthetized and perfused through the ascending aorta with isotonic saline, followed by 4% paraformaldehyde. Then, spinal cord, substantia nigra, sciatic nerves and DRGs were post-fixed in 4% paraformaldehyde, dehydrated with grade sucrose solution and included in optimal cutting temperature compound (OCT, Sakura Finetek). Tissue was sectioned using a cryostat (Leica) and mounted on Superfrost Plus slides (Thermo Fisher Scientific). DRG and sciatic nerve were transversally or longitudinally sectioned at 10 μm, substantia nigra was coronally sectioned at 25 μm and spinal cord from thoracic vertebrae T11-T13 was sectioned transversally at 12 μm.

For IF, sections were blocked/permeabilized with 2% fish skin gelatin (Sigma-Aldrich) and 0.1% Triton X-100 and incubated with primary antibodies in the same solution. Immunoreactive proteins were visualized with fluorophores-conjugated secondary antibodies. Samples were mounted in Vectashield with DAPI (Vector Laboratories) as previously described^14^. Sections were immunostained using the following antibodies: chicken anti-neurofilament medium chain (NF-M), 1:1000, (#AB5753, Millipore Bioscience Research Reagents); rabbit anti-myelin basic protein (MBP), 1:500, (M3821, Sigma); rat anti-Cd11b, 1:500, (MCA74G, Serotec); mouse anti-KDEL, 1:250, (SPA-827, Stressgen) and rabbit anti-S-100, 1:400 (Z0311, Dako). For IHC of CHOP, tissue was incubated with 3% H_2_O_2_/10% methanol, followed by epitope retrieval using buffer citrate pH 6.0 (Dako). Sections were incubated in blocking solution (10% Goat serum, 4% BSA, 0.1% Triton X-100) and then incubated with rabbit anti-CHOP antibody, 1:100 (Sc-7351, Santa Cruz) ON at 4 °C. Immunoreactivity was developed with DAB HRP substrate kit (Vector Laboratories). Images were obtained in an inverted Olympus IX7I fluorescent microscope equipped with a 40X objective (Olympus LUCPlanFLN NA 0.60 Ph2, Olympus). Quantification of Cd11b^+^ macrophages and double positive NF-M/GFP axons was represented as density (number of cells/axons per area) in at least 3 pictures per mice.

For EM analysis, sciatic nerves were fixed overnight with 2.5% glutaraldehyde, 0.01% picric acid and 0.05 M cacodylate buffer, pH 7.3. Nerves were incubated in the same buffer with 1% OsO_4_ and then immersed in 2% uranyl acetate, dehydrated in a gradient of ethanol and acetone, and infiltrated in Epon (Ted Pella) as previously described^29^. Transversal semi-thin and ultrathin sections were obtained using an ultramicrotome (Reichert). Thin 80 nm sections were obtained and mounted in copper grids and contrasted using 1% uranyl acetate and lead citrate. Observations of the grids were made using a Phillips Tecnai 12 (Eindhoven) at 80 KV and photographed by a Mega view G2 camera (Olympus). Quantification of 1 μm semi-thin slices were made to identify intact fibers and remyelinated axons by size, axoplasm content, and condensed myelin. Degenerated myelins were identified by decompacted myelin and aberrant compaction of myelin morphology. Total number of remyelinated axons and degenerated myelins were normalized by area in 3 regions of the distal nerve.

Quantification of the g-ratio was performed by measuring the area of the axoplasm and the area of the complete fiber including the myelin sheath. Then, axonal and fiber diameters were calculated assuming circular structures. At least 80 fibers for each mouse strain were analyzed by EM. Nerve area measurement was performed in the biggest fascicle in 3 animals per genotype. All images were analyzed using ImageJ software (NIH).

### AAV injection in dorsal root ganglia

The production and quantification of recombinant AAV2.XBP1s/GFP (2.9 × 10^12^ DRP/ml), AAV2.GFP (1.22 × 10^12^ DRP/ml) and AAV2.shXBP1 (3.5 × 10^11^ DRP/ml) was described before^14^,^27^. For DRG gene transfer, C57BL6 mice containing 6 lumbar vertebrae were used. In this mouse strain, L3 and L4 spinal nerves provide the major contribution of sciatic nerve axons, and the L5 spinal nerve have a smaller contribution^28^.

Mice were anesthetized and skin and muscles were separated exposing the lateral region of right third lumbar (L3) and L4 vertebrae and the dorsal region transverse processes. Then, a right hemi-laminectomy was performed in L3 and L4 to remove the transverse processes. For each DRG, 1 μl of the AAV mixed with 0.3% fast green was injected at a flow of 0.01 μl/s using a microinjector (Neurostar InjectoMate, IM1A193) and a 2 μl Hamilton syringe fitted with a 34G needle. 7 days after injection, the sciatic nerve was injured at the notch level. To evaluate axonal regeneration, a 3 mm segment located 3 mm distal or 6 mm proximal to the crush site was removed at 14 dpi for immunofluorescence analysis.

### Statistical analysis

Data are shown as mean ± SEM. Statistical analysis were performed using repeated measure ANOVA, followed by a Bonferroni post hoc test and Student’s t-test and analyzed using GraphPad Prims 5 software.

## Additional Information

**How to cite this article**: Oñate, M. *et al*. Activation of the unfolded protein response promotes axonal regeneration after peripheral nerve injury. *Sci. Rep*. **6**, 21709; doi: 10.1038/srep21709 (2016).

## Supplementary Material

Supplementary Information
