# Author Correction: Activation of the unfolded protein response promotes axonal regeneration after peripheral nerve injury

**DOI:** 10.1038/s41598-021-04003-2

**Published:** 2021-12-20

**Authors:** Maritza Oñate, Alejandra Catenaccio, Gabriela Martínez, Donna Armentano, Geoffrey Parsons, Bredford Kerr, Claudio Hetz, Felipe A. Court

**Affiliations:** 1 Geroscience Center for Brain Health and Metabolism, Santiago, Chile; 2 Millenium Nucleus for Regenerative Biology, Faculty of Biology, Pontificia Universidad Católica de Chile, Santiago, Chile; 3 Biomedical Neuroscience Institute, Faculty of Medicine, University of Chile, Santiago, Chile; 4 Program of Cellular and Molecular Biology, Institute of Biomedical Sciences, Center for Molecular Studies of the Cell, University of Chile, Santiago, Chile; 5 Department of Molecular Biology, Genzyme Corporation, 49 New York Avenue, Framingham, MA 01701 USA; 6 Centro de Estudios Científicos, Valdivia, Chile; 7 Department of Immunology and Infectious Diseases, Harvard School of Public Health, Boston, MA USA

Correction to: *Scientific Reports* 10.1038/srep21709, published online 24 February 2016

The original version of this Article contains errors in the microscopy images in Figure 6, where parts of the image for “Uninjured/ XP1Nes-/-” in panel A was used in error to create the images for “Uninjured / Non-Tg” and “Uninjured / Tg XBPS1s” in panel B.

The correct Figure 6 and accompanying legend appear below.

**Figure 6:**
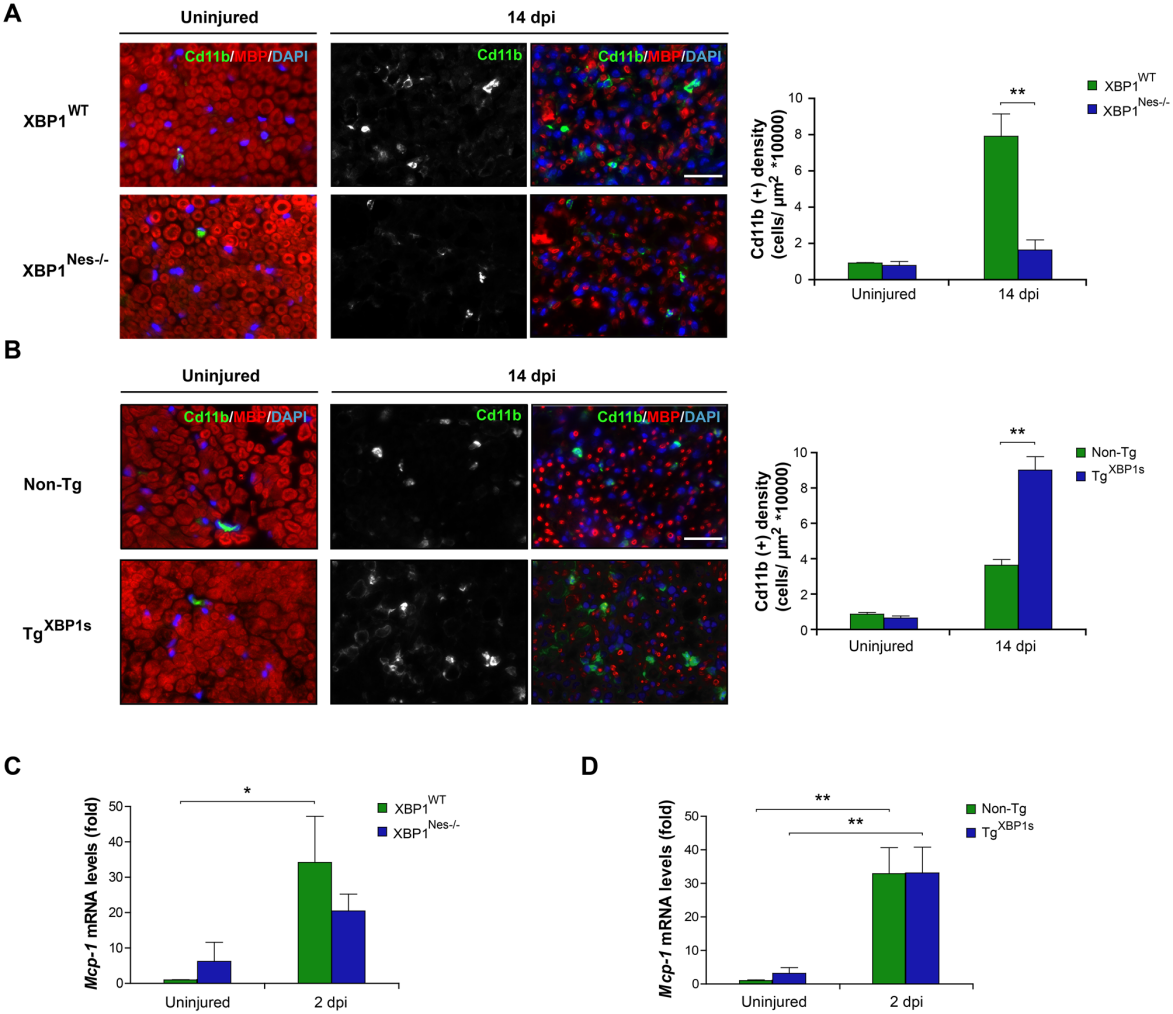
XBP1 expression in the nervous system enhances macrophage infiltration in injured sciatic nerves. **(A)** Sciatic nerves from XBP1^Nes−/−^ and XBP1^WT^ littermates were processed for immunofluorescence from uninjured conditions and at 14 dpi distal sciatic nerves were analyzed for Cd11b (green) to evaluate macrophages and MBP (red) to stain myelin sheaths. Nuclei were counterstained using DAPI (blue, left panel). The staining density for Cd11b was quantified at 14 dpi in XBP1^Nes−/−^ and XBP1^WT^ mice (right panel). **(B)** Tg^XBP1s^ and non-Tg sciatic nerves were analyzed as described in A. *Mcp-1* expression was analyzed in sciatic nerves of XBP1^Nes−/−^ and XBP1^WT^ mice **(C)** or in Tg^XBP1s^ and non-Tg sciatic nerves **(D)** by real-time PCR at 2 dpi. Data are shown as mean ± S.E.M.; *p < 0.05; **p < 0.01. Data were analyzed by student’s t-test at each time point (n = 3 animals per group). Scale bar: 20 μm.

