# Supplementary material for: Activation of the unfolded protein response promotes axonal regeneration after peripheral nerve injury
Source: Sci Rep. 2016 Feb 24;6:21709. doi: 10.1038/srep21709 (PMC4764858; doi:10.1038/srep21709)

Activation of the unfolded protein response promotes axonal regeneration after peripheral nerve injury

Maritza Oñate, Alejandra Catenaccio, Gabriela Martínez, Donna Armentano, Geoffrey Parsons, Bredford Kerr, Claudio Hetz and Felipe A. Court

Online Supplementary material

Supplementary Figure S1. *Chop* and *Gadd34* are not upregulated after sciatic nerve crush. (A) *Chop* and **(B)** *Gadd34* expression were analyzed from sciatic nerves by real-time PCR in uninjured conditions and at 2 dpi in distal nerve segments. **(C)** CHOP expression was evaluated by immunohistochemistry in wild-type mice at 8 dpi in the spinal cord (left panel) and DRGs (upper right panel). As a positive control, wild-type animals were unilaterally injected with tunicamycin (Tm) in the right substantia nigra for 24 hours (lower right panel). Data are expressed as mean ± S.E.M.; Student's t-test (n = 3 animals per group). Scale bars: Spinal cord, 100 μm (low magnification) and 40 μm (high magnification); DRG, 100 μm; substantia nigra, 500 μm (low magnification) and 20 μm (high magnification).

Supplementary Figure S2. *Cre* and *Xbp1s* mRNA expression in XBP1$^{Nes-/-}$ and XBP1s^{Tg} mice. (A) *Cre recombinase* expression was analyzed in cerebellum, DRGs and sciatic nerves of XBP1$^{Nes-/-}$ and XBP1^{WT} mice by real-time PCR in uninjured conditions. **(B)** The deleted exon II of the *Xbp1* gene was measured in DRGs and sciatic nerves of XBP1$^{Nes-/-}$ and XBP1^{WT} mice by real-time PCR in uninjured conditions. **(C)** *Xbp1s* mRNA expression was analyzed by real-time PCR in Tg^{XBP1s} and non-Tg mice in uninjured conditions in cerebellum, DRGs and sciatic nerve. In all panels, data are shown as mean ± S.E.M.; *: $p < 0.05$; **: $p < 0.01$; ***: $p < 0.001$. Statistical differences were analyzed by student's t-test (n = 3 animals per group).

Supplementary Figure S3. Overexpression of XBP1s increases myelin removal, axonal regeneration and macrophage infiltration after nerve injury. (A) Transverse semi-thin sections of sciatic nerves from uninjured and 11 dpi Tg^{XBP1s} and Non-Tg mice stained with toluidine blue. Sections were obtained 3 mm distal to crush region. Black and white arrowheads indicate demyelinated and regenerated fibers, respectively (left panel). Quantification on the number of degenerated myelins and remyelinated fibers per area from transverse semi-thin sections of each mouse strain (right panel). Scale bar: 10 μm. **(B)** Distal sciatic nerves from Tg^{XBP1s} and Non-Tg animals were analyzed for Cd11b (green) at 5 dpi and in uninjured conditions. Nuclei were stained using DAPI (blue) (left panel). Quantification of $Cd11b^{+}$ density of each genotype (right panel). Scale bar: 20 μm. Data are shown as mean ± S.E.M.; *: $p < 0.05$, **: $p < 0.01$. Morphological data were analyzed by student´s t-test at each time point (n = 3 animals per group).

Supplementary Figure S4. Modulation of XBP1 does not affect remyelination after nerve injury. (A) Electron microscopy of $XBP1^{Nes-/-}$ and $XBP1^{WT}$ distal nerves at 14 dpi. g-ratio was measure as the ratio of axonal diameter (d) to the fiber diameter (axon and myelin sheath) (D, left panel). Scatter plot of g-ratio values of $XBP1^{Nes-/-}$ and $XBP1^{WT}$ remyelinated axons (right panel). **(B)** Tg^{XBP1s} and Non-Tg mice were analyzed as in A. Scale bar: 2 μm.

Supplementary Figure S5. Modulation of XBP1 do not affect nerve area after injury. (A) Nerve area of $XBP1^{Nes-/-}$ and $XBP1^{WT}$ was measured in uninjured conditions and at 14 dpi in optic semi-thin slices. **(B)** Tg^{XBP1s} and Non-Tg mice were analyzed as in A. Data are shown as ± S.E.M. Statistical differences were evaluated by student´s t-test at each time point (n = 3 animals per group).

Supplementary Figure S1

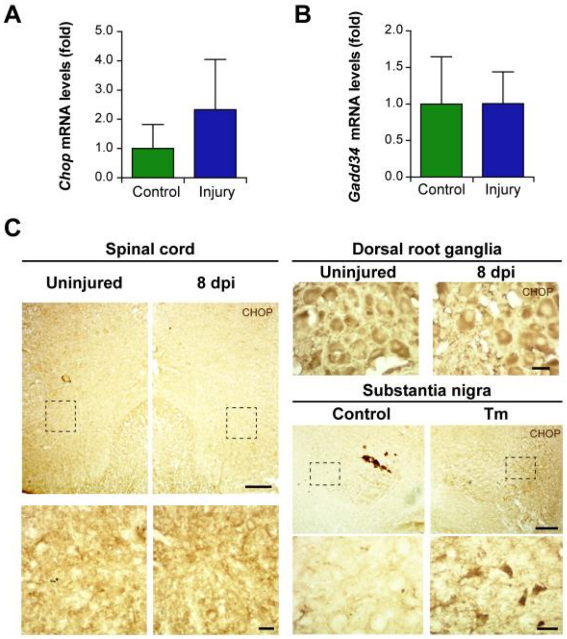

Supplementary Figure S2

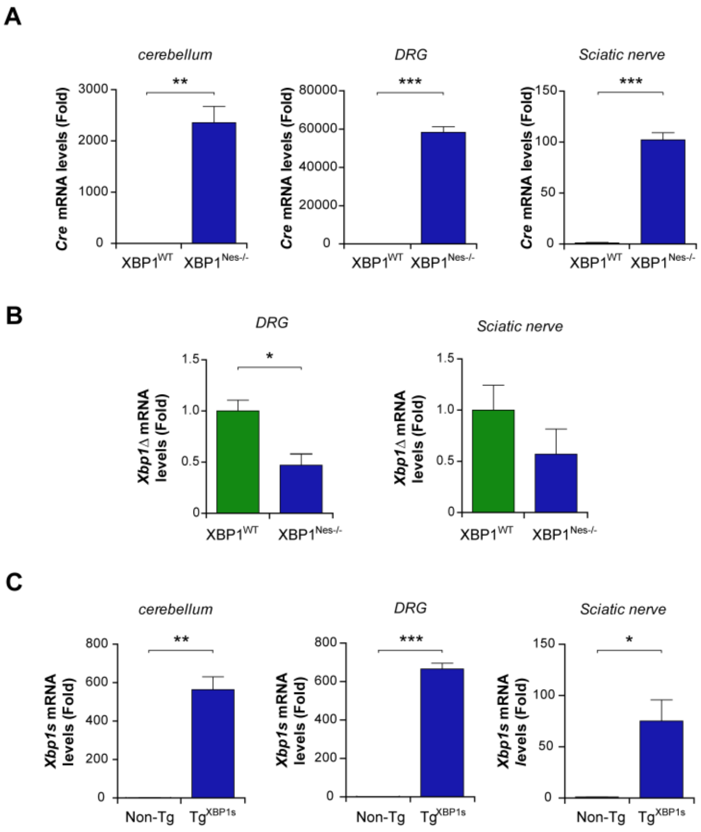

Supplementary Figure S3

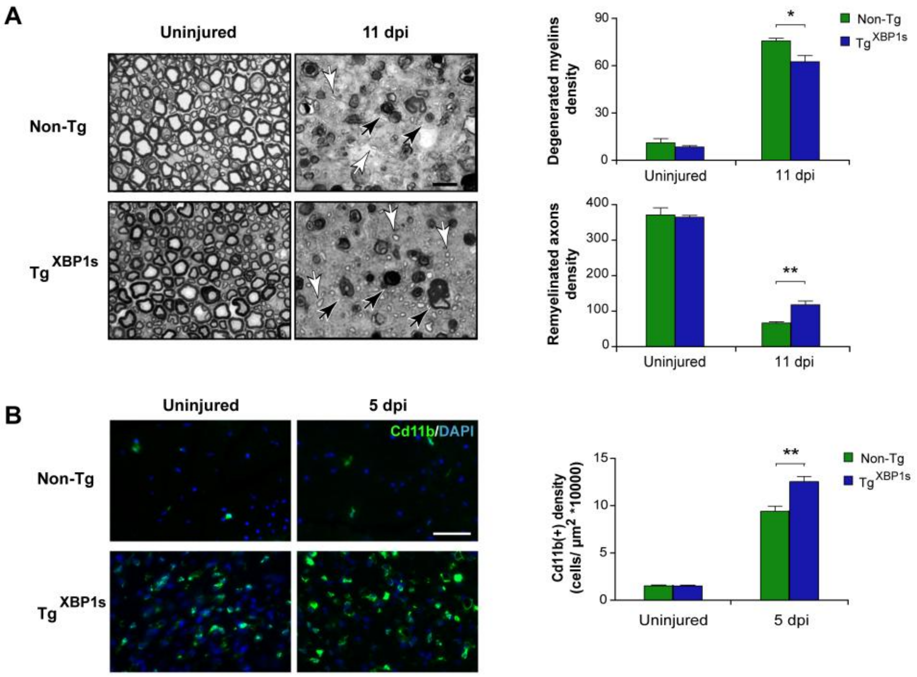

Supplementary Figure S4

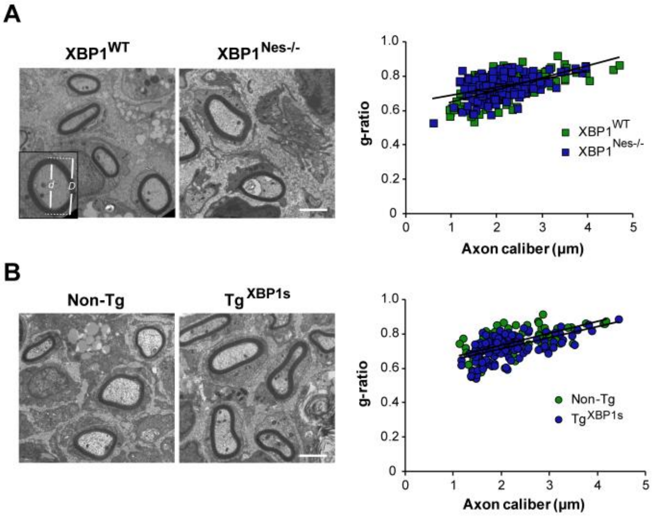

Supplementary Figure S5

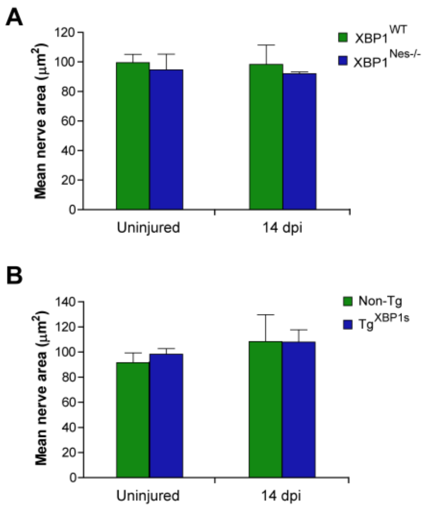

Supplement: Supplementary Information [file srep21709-s1.pdf]
